# Bilateral dual iliac screw pelvic fixation for adult spinal deformity: a case report of a superior gluteal artery pseudoaneurysm secondary to aberrant iliac screw trajectory

**DOI:** 10.1007/s43390-023-00774-y

**Published:** 2023-10-26

**Authors:** Monty Khela, Rafid Kasir, R. Peter Lokken, Aaron J. Clark, Alekos A. Theologis

**Affiliations:** 1 School of Medicine, Creighton University, Omaha, NE USA; 2 Department of Orthopaedic Surgery, University of California-San Francisco (UCSF), 500 Parnassus Ave, MUW 3rd Floor, San Francisco, CA 94143 USA; 3 Department of Radiology and Biomedical Imaging, UCSF, San Francisco, CA USA; 4 Department of Neurological Surgery, UCSF, San Francisco, CA USA

**Keywords:** Adult spinal deformity, Pelvic fixation, Pseudoaneurysm, Endovascular embolization, Superior gluteal artery

## Abstract

**Purpose:**

To present a case of a pseudoaneurysm of a branch of the left superior gluteal artery (SGA) secondary to lateral wall perforation from an iliac screw and its subsequent evaluation and management.

**Methods:**

Case report.

**Results:**

A 67-year-old female with a history of degenerative flatback and scoliosis and pathological fractures of T12 and L1 secondary to osteodisciitis underwent a single0stage L5–S1 ALIF and T9-pelvis posterior instrumented fusion with bilateral dual iliac screw fixation, revision T11–S1 decompression, and T12 and L1 irrigation and debridement and partial corpectomies. During the operation, non-pulsatile bleeding was encountered after creating an initial trajectory for the more proximal of the two left iliac screws. While the initial post-operative course was benign, the patient was readmitted for hypotension and anemia. Computed tomography of the abdomen/pelvis demonstrated a pseudoaneurysm (2.3 cm × 2.1 cm × 2.3 cm) of a branch of the left SGA. Diagnostic angiogram confirmed a pseudoaneurysm off of one of the branches of the left SGA. Endovascular embolization using multiple coils resulted in a complete cessation of blood flow in the pseudoaneurysm. At 2 years follow-up, no symptoms suggestive of recurrence of the pseudoaneurysm were reported.

**Conclusions:**

A pseudoaneurysm of a branch of the left superior gluteal artery as a result of lateral wall perforation from an aberrantly placed iliac screw during an adult spinal deformity operation involving dual screw pelvic fixation is reported. Prompt recognition, multidisciplinary collaboration, and appropriate intervention were key in achieving a successful outcome and preventing further morbidity.

## Introduction

Pelvic fixation has proven critical for protecting sacral fixation and decreasing complications at the lumbosacral junction in adult spinal deformity (ASD) operations [1–4]. While iliac screws are generally safe, they can be associated with complications, including neurovascular injury [5, 6]. In this report, we present a case of a pseudoaneurysm of a branch of the left superior gluteal artery (SGA) secondary to lateral wall perforation from an iliac screw.

## Case presentation

**Fig. 1:**
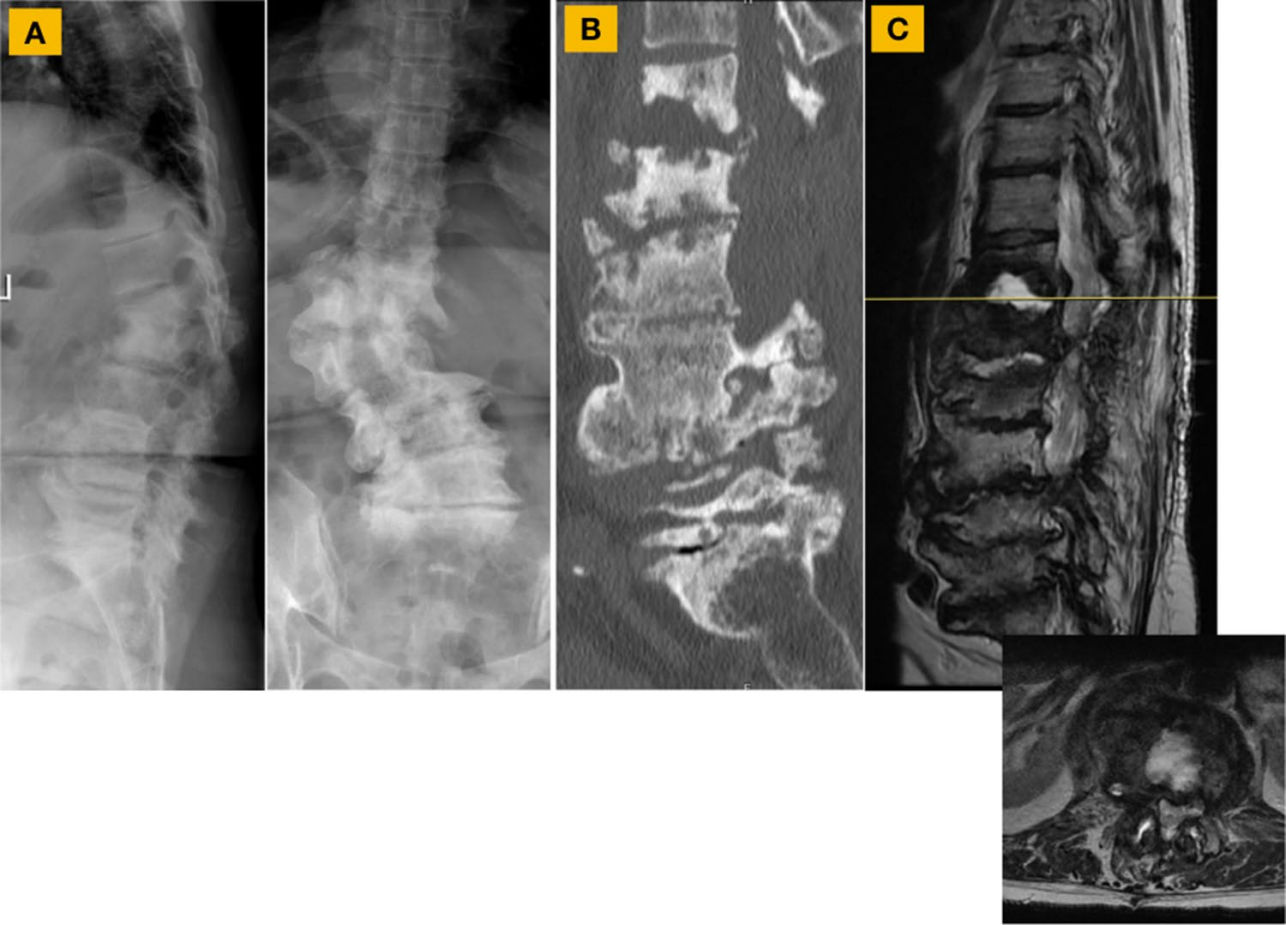
**A** Presenting radiographs demonstrated degenerative scoliosis, flatback, and pathological vertebral body fractures with posterior element subluxation at T12–L1 resulting in thoracolumbar kyphosis. **B** Computed Tomography (CT) confirmed the T12 and L1 pathological fractures but also showed flexibility of the segmental kyphosis. **C** Magnetic resonance imaging of the lumbar spine revealed severe central stenosis at T12–L1, severe lateral recess and foraminal stenosis from T12–S1 bilaterally, and a prior narrow T12–L4 decompression

A 67-year-old female presented with back pain, leg pain, leg weakness, and difficulty standing upright in the setting of a prior T12–L4 laminectomy for epidural abscess and subsequent development of T12–L1 osteodisciitis (*Gemella Morbillorum*). Imaging demonstrated degenerative scoliosis, flatback, pathological vertebral body fractures at T12–L1, and central stenosis at T12–L1 (Fig. 1).

**Fig. 2:**
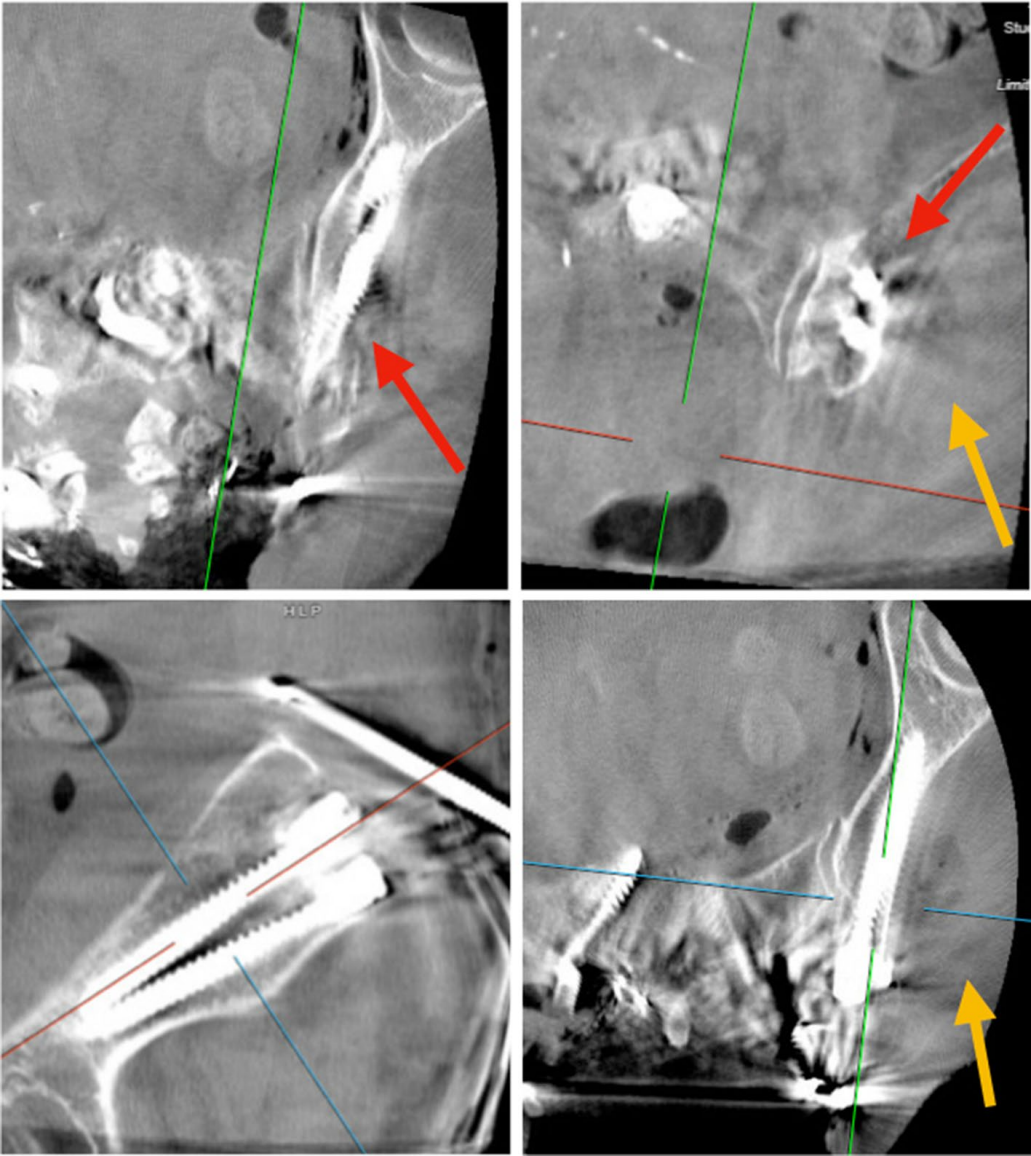
While intra-operative CT scan demonstrated appropriately re-positioned iliac screws, visible were the cortical breach of the lateral wall of the ilium (red arrows) as well as hypointense fluid (gold arrows), likely representing blood, from the originally aberrantly planned trajectory for the proximal iliac screw

A L5–S1 anterior lumbar interbody fusion and T9-pelvis posterior instrumented fusion, revision decompression from T11 to S1, and partial corpectomies of T12 and L1 were performed. For pelvic fixation, two iliac bolts were placed bilaterally to facilitate the construction of a multi-rod construct. Both iliac screws on the right and the most distal screw on the left were placed without incident. The second (more proximal) iliac screw’s trajectory on the left was cannulated with a gearshift, which was felt to be all intraosseous. The trajectory was then tapped. Upon removal of the tap, more than expected, brisk, non-pulsatile blood egressed from the ilium. Palpation of the trajectory confirmed a breach of the ilium’s outer table. Gelfoam powder was injected into the hole and the opening was covered with bone wax. A more medial trajectory was created with a gearshift. This new trajectory was tapped and an 8.0 × 90 mm screw was placed. Subsequent intra-operative CT scan showed appropriately positioned iliac screws (Fig. 2).

**Fig. 3:**
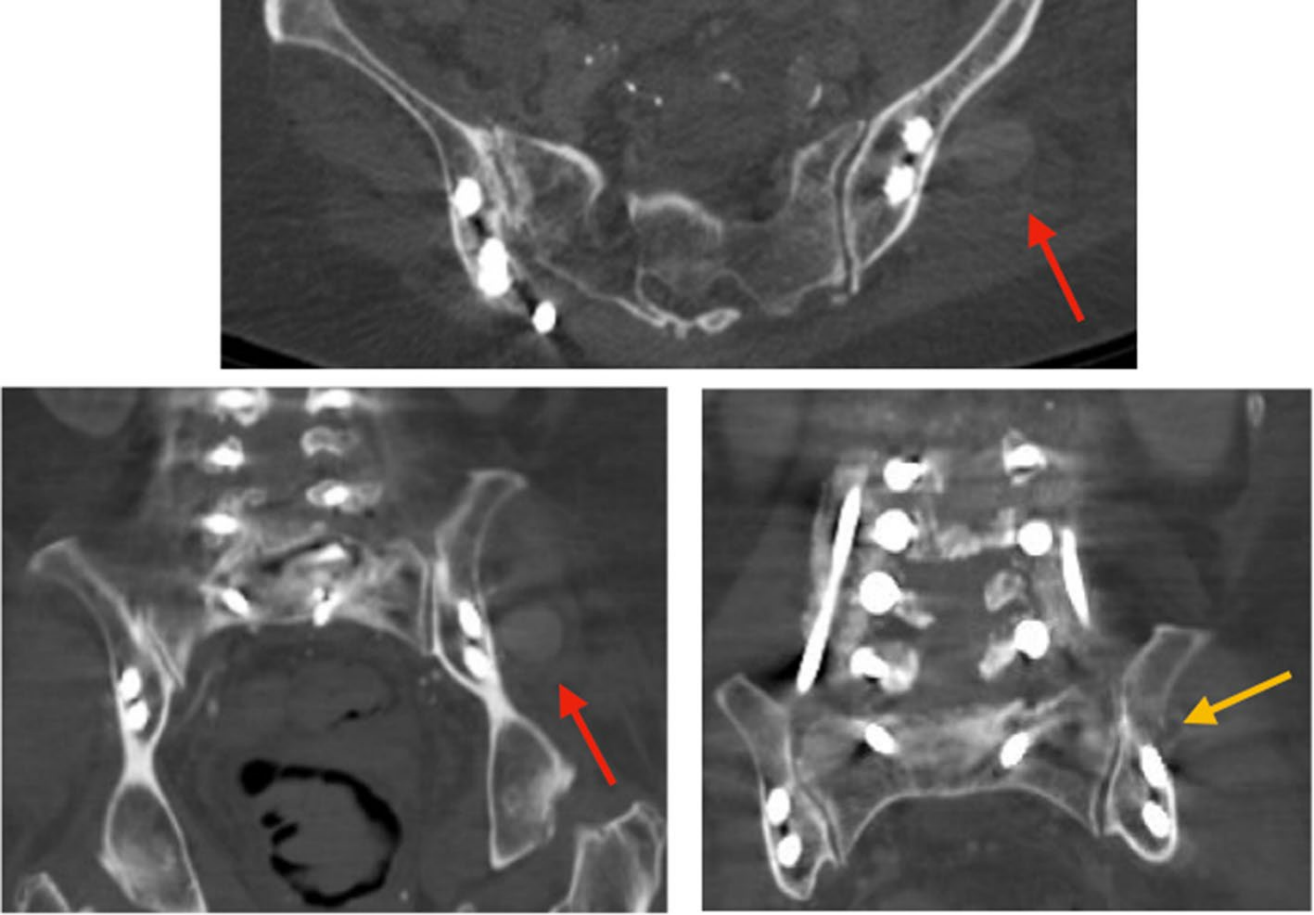
A CT abdomen/pelvis with contrast obtained during re-admission demonstrated an abnormal rounded focus of contrast enhancement adjacent to the left iliac bone measuring up to 2.3 cm × 2.1 cm × 2.3 cm (red arrows). Note the cortical breach of the lateral wall of the ilium (gold arrow) from the original aberrantly planned trajectory for the proximal iliac screw

While the initial post-operative course was uneventful, she was readmitted 2 weeks post-op for hypotension. CT angiogram of the abdomen/pelvis reported an abnormal rounded focus of contrast enhancement adjacent to the left iliac bone measuring up to 2.3 × 2.1 × 2.3 cm, which was consistent with a pseudoaneurysm (Fig. 3).

**Fig. 4:**
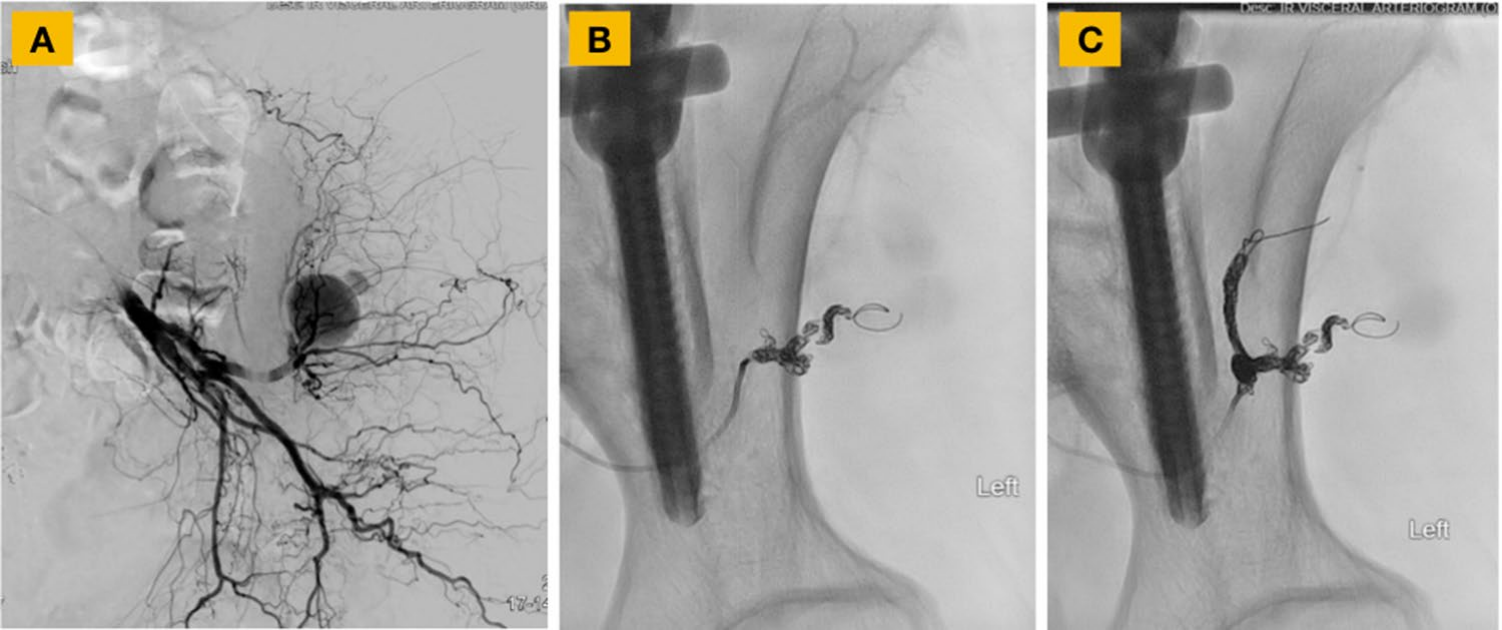
During angiogram, a pseudoaneurysm off of one of the branches of the left superior gluteal artery was found (**A**). Embolization within the pseudoaneurysm using Gelfoam and coils was first performed (**B**), followed by embolization using additional coils more proximal to the pseudoaneurysm (**C**), which resulted in complete cessation of blood flow in the pseudoaneurysm (**C**)

Angiogram of the left internal iliac, left SGA, and branches of the left SGA was performed (Fig. 4). A pseudoaneurysm off a branch of the left SGA was found (Fig. 4). Embolization within the pseudoaneurysm and proximal to the pseudoaneurysm using Gelfoam and 7 coils resulted in complete cessation of the pseudoaneurysm’s blood flow (Fig. 4). Two days later, the patient was discharged.

**Fig. 5:**
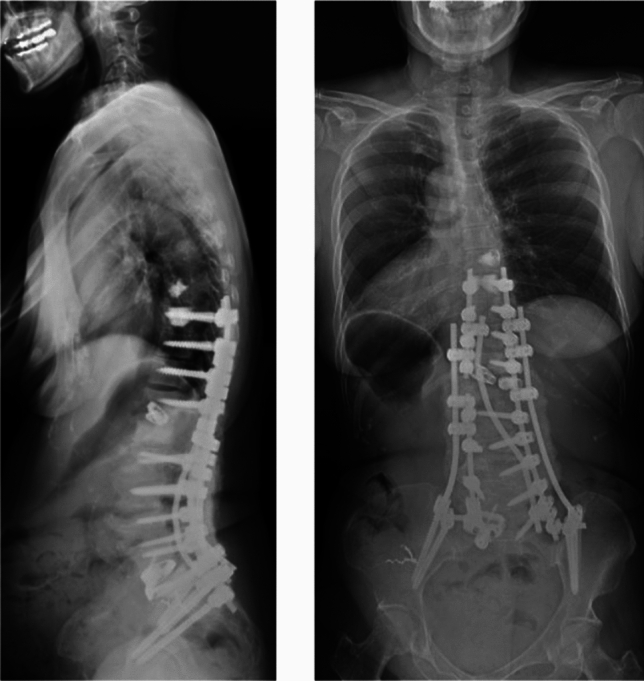
Post-operative radiographs demonstrated improved and acceptable regional and global coronal and sagittal spinal alignment

At 2 years post-operatively, the patient had significantly improved back pain, leg pain and weakness, and posture (Fig. 5). No recurrence of the pseudoaneurysm was reported at 2 years follow-up.

## Discussion

The SGA is a blood vessel that arises from the internal iliac artery within the pelvis. Once the SGA emerges from the greater sciatic notch, it branches into its superficial and deep branches and courses over the outer table of the pelvis, often running in a groove located on the iliac side of the posterior superior iliac spine (PSIS) [7].

Injury to the SGA is a well-known phenomenon from iliosacral screws and trans-sacral screws from outside-in trajectories to stabilize pelvic/sacral/SI joint injuries [8–10]. In spine surgery, SGA injuries have been reported secondary to iliac crest bone graft harvest from the PSIS [11–14]. The SGA’s location on the pelvis’ outer table puts it at risk from aberrantly placed iliac and S2AI screws, particularly ones that breach the ilium’s lateral wall more cranially [7]. In our patient, the disruption of one of the SGA’s branch occurred secondary to an iliac screw trajectory that perforated the ilium’s lateral cortex. This screw was the more proximal of the two iliac screws as part of a dual iliac fixation technique. The more proximal start-point of the screw increased its risk of a lateral wall perforation given a narrow osseous corridor more cranially in the iliac wing. To minimize this occurrence, an iliac screw with a more proximal start point ideally should be aimed more distally so as to enter the supra-acetabular osseous corridor.

Pseudoaneurysms may present with a palpable mass, pulsatile bleeding, or compressive symptoms. They can be managed conservatively or with endovascular techniques, including embolization, stenting, or coiling. The choice of treatment depends on the size, location, and clinical presentation of the lesion. In our patient, embolization through an endovascular approach was utilized with good success.

## Conclusion

This case report highlights the occurrence of a pseudoaneurysm of a branch of the left SGA secondary to lateral wall perforation from an aberrantly placed iliac screw during an adult spinal deformity operation. Prompt recognition, multidisciplinary collaboration, and appropriate intervention were key in achieving a successful outcome and preventing further morbidity.

## Data Availability

Data available on request from the authors.
